# Rapid Detection of an ABT-737-Sensitive Primed for Death State in Cells Using Microplate-Based Respirometry

**DOI:** 10.1371/journal.pone.0042487

**Published:** 2012-08-03

**Authors:** Pascaline Clerc, Gregory B. Carey, Zara Mehrabian, Michael Wei, Hyehyun Hwang, Geoffrey D. Girnun, Hegang Chen, Stuart S. Martin, Brian M. Polster

**Affiliations:** 1 Department of Anesthesiology and the Shock, Trauma and Anesthesiology Research Center, University of Maryland School of Medicine, Baltimore, Maryland, United States of America; 2 Department of Microbiology and Immunology, University of Maryland School of Medicine, Baltimore, Maryland, United States of America; 3 Department of Biochemistry and Molecular Biology, University of Maryland School of Medicine, Baltimore, Maryland, United States of America; 4 Department of Epidemiology and Public Health, University of Maryland School of Medicine, Baltimore, Maryland, United States of America; 5 Department of Physiology, University of Maryland School of Medicine, Baltimore, Maryland, United States of America; Boston University, United States of America

## Abstract

Cells that exhibit an absolute dependence on the anti-apoptotic BCL-2 protein for survival are termed “primed for death” and are killed by the BCL-2 antagonist ABT-737. Many cancers exhibit a primed phenotype, including some that are resistant to conventional chemotherapy due to high BCL-2 expression. We show here that 1) stable BCL-2 overexpression alone can induce a primed for death state and 2) that an ABT-737-induced loss of functional cytochrome *c* from the electron transport chain causes a reduction in maximal respiration that is readily detectable by microplate-based respirometry. Stable BCL-2 overexpression sensitized non-tumorigenic MCF10A mammary epithelial cells to ABT-737-induced caspase-dependent apoptosis. Mitochondria within permeabilized BCL-2 overexpressing cells were selectively vulnerable to ABT-737-induced cytochrome *c* release compared to those from control-transfected cells, consistent with a primed state. ABT-737 treatment caused a dose-dependent impairment of maximal O_2_ consumption in MCF10A BCL-2 overexpressing cells but not in control-transfected cells or in immortalized mouse embryonic fibroblasts lacking both BAX and BAK. This impairment was rescued by delivering exogenous cytochrome *c* to mitochondria via saponin-mediated plasma membrane permeabilization. An ABT-737-induced reduction in maximal O_2_ consumption was also detectable in SP53, JeKo-1, and WEHI-231 B-cell lymphoma cell lines, with sensitivity correlating with BCL-2:MCL-1 ratio and with susceptibility (SP53 and JeKo-1) or resistance (WEHI-231) to ABT-737-induced apoptosis. Multiplexing respirometry assays to ELISA-based determination of cytochrome *c* redistribution confirmed that respiratory inhibition was associated with cytochrome *c* release. In summary, cell-based respiration assays were able to rapidly identify a primed for death state in cells with either artificially overexpressed or high endogenous BCL-2. Rapid detection of a primed for death state in individual cancers by “bioenergetics-based profiling” may eventually help identify the subset of patients with chemoresistant but primed tumors who can benefit from treatment that incorporates a BCL-2 antagonist.

## Introduction

BCL-2 is an anti-apoptotic protein that was discovered by mapping a t(14;18) chromosomal translocation in B-cell lymphoma that results in BCL-2 overexpression [1], [2]. Overexpression of BCL-2 by cancer cells promotes survival during tumorigenesis and can lead to acquired chemoresistance [3]–[5]. BCL-2 and related anti-apoptotic members inhibit apoptosis by sequestering “activator” BH3-only proteins (e.g. BID or BIM) that signal pro-apoptotic conformational changes in the multi-domain BCL-2 family proteins BAX or BAK [6], [7]. BCL-2 may also bind and directly inhibit active conformations of BAX and BAK [7], [8]. Sensitizer BH3-only proteins (e.g. NOXA or BAD) decrease the threshold for BAX/BAK activation by binding anti-apoptotic BCL-2 proteins and preventing them from sequestering “activator” molecules. BAX/BAK-mediated mitochondrial outer membrane permeabilization occurs when activator BH3-only proteins at the mitochondria exceed the inhibitory capacity of anti-apoptotic BCL-2 family members that include BCL-2, BCL-xL, BCL-W, MCL-1, and BFL-1/A1 [3], [9], [10]. Outer membrane permeabilization leads to cytochrome *c* (cyt *c*) redistribution to the cytoplasm which initiates caspase-dependent apoptosis [11], [12]. Cells are defined as “primed for death” if inhibition of anti-apoptotic BCL-2 family proteins alone, such as with the BH3 mimetic ABT-737 [13], is sufficient to release cyt *c* and drive apoptosis [14].

**Figure 1:**
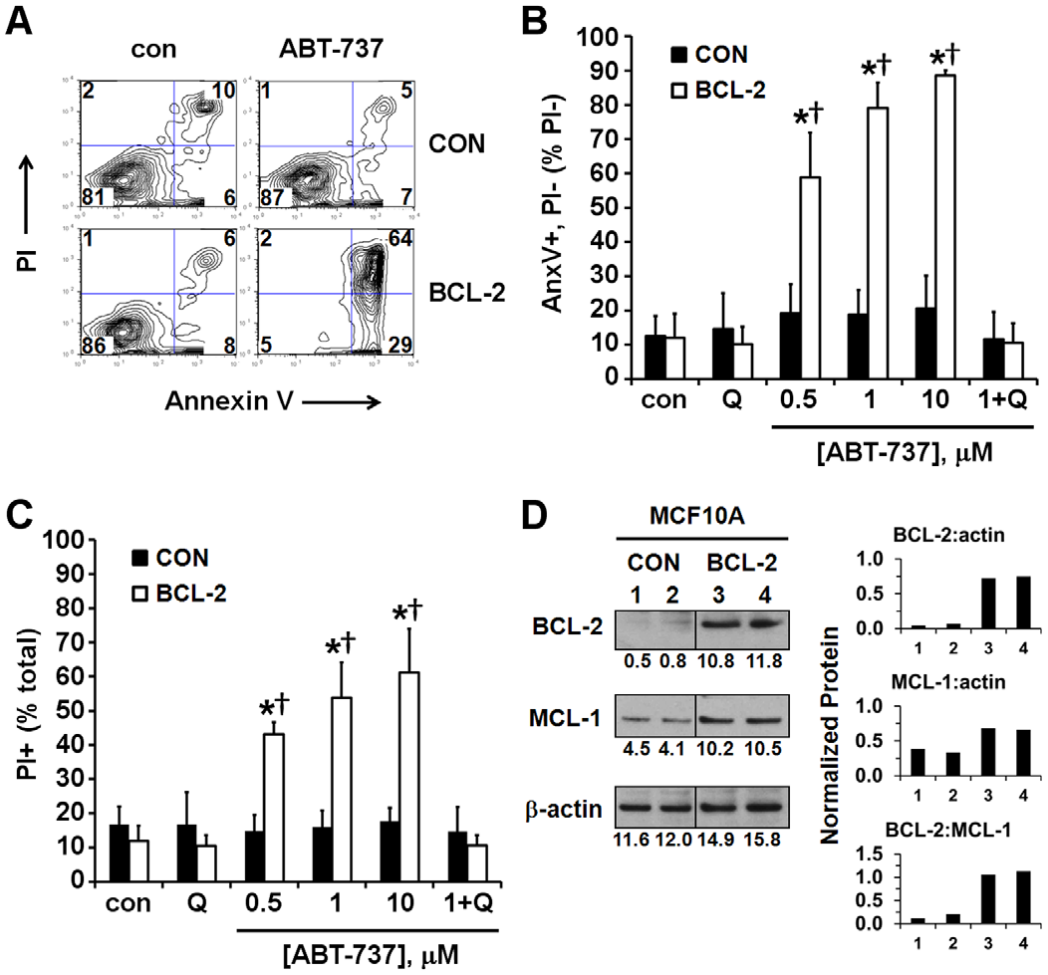
Stable BCL-2 overexpression in MCF10A cells induces an ABT-737-sensitive primed for death state. (A) Representative data from a flow cytometry experiment to determine the percentage of control-transfected (MCF10A CON) or BCL-2 overexpressing (MCF10A BCL-2) cells stained positively with Annexin V (AnxV), propidium iodide (PI), or both following a 4 h treatment with vehicle (con) or ABT-737 (10 µM). Numbers indicate the percentage of cells in the gated populations. Cells to the right of the vertical gate were considered AnxV positive and cells above the horizontal gate were considered PI positive. (B) Early apoptosis, expressed as the percentage of PI negative cells (bottom two quadrants in A) that were AnxV-positive (bottom right quadrant) after 4 h of treatment with vehicle control (con), ABT-737, Q-VD (Q, 20 µM), or ABT-737+ QVD. (C) Cell death, expressed as percentage of PI positive cells after 4 h of the treatments in B. Results in B and C are mean ± SE of three experiments performed in triplicate. *p<0.05 for ABT-737-treated relative to control-treated; †p<0.05 for MCF10A BCL-2 relative to control-transfected cells. (D) Expression levels of BCL-2 and MCL-1 relative to β-actin loading control in MCF10A CON and BCL-2 cells. Numbers below protein bands are optical density values. Protein normalized to β-actin and BCL-2:MCL-1 ratios are plotted in the right panel for lanes 1–4.

ABT-263 (Navitoclax), the orally bioavailable analogue of ABT-737, is in clinical trials for chronic lymphocytic leukemia, lymphoma, and small cell lung cancer [3], [15]–[18]. ABT-263/ABT-737 is also in preclinical testing for a number of additional cancer types including breast cancer [19], [20]. However, ABT-737 does not exhibit efficacy against all tumor types and the responsiveness within a given tumor type is heterogeneous. The factors determining whether an ABT-737-sensitive primed for death state exists are complex and depend on the state of multiple BCL-2 family members including both ABT-737 sensitive (e.g. BCL-2) and resistant (e.g. MCL-1) anti-apoptotic molecules, “activator” (e.g. BIM) and “sensitizer” (e.g. NOXA) BH3-only proteins, and multi-domain pro-apoptotic proteins BAX and BAK [3], [21]. Some studies have found an association between MCL-1 levels and ABT-737 sensitivity while others have found a stronger association with BCL-2 levels [19], [22]–[24]. Improving our ability to stratify patients for treatment by developing more robust predictive biomarkers would promote the success of BCL-2 inhibitors in clinical trials and guide their future use in cancer therapy. Evidence suggests that identification of a primed for death state may also help predict the response of tumors to in-use chemotherapeutics [25]. Because individual protein biomarkers have not consistently predicted ABT-737 sensitivity, we sought a functional approach that, once optimized for human tumor tissue, might eventually accomplish this goal.

**Figure 2:**
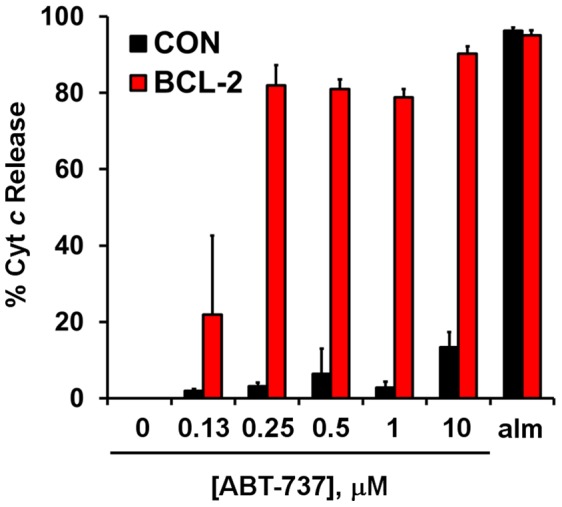
MCF10A BCL-2 mitochondria are selectively sensitive to ABT-737-induced cytochrome *c* release. Cyt *c* was quantified by ELISA in the medium and cell lysate after a 28 min incubation of permeabilized cells with vehicle, ABT-737 (as indicated), or alamethicin (alm, 80 µg/ml). Succinate (5 mM, in the presence of rotenone, 0.5 µM), ADP (1 mM), and K_2_HPO_4_ (3.6 mM) were present to support mitochondrial respiration during the treatment. Results are expressed as % cyt *c* released into the medium compared to the total cyt *c* that was quantified (mean ± SD, n = 3). Cyt *c* was not detectable in the medium of permeabilized cells treated with vehicle.

One possible approach is a bioenergetics-based approach, as we and others previously demonstrated that cyt *c* release can limit mitochondrial O_2_ consumption due to depletion of cyt *c* from the electron transport chain [26]–[29]. Here, we first tested the hypothesis that stable BCL-2 overexpression alone can induce a primed for death state in ABT-737-resistant non-tumorigenic MCF10A mammary epithelial cells, providing a convenient model to distinguish primed from unprimed cells. Second, we used microplate-based respirometry to evaluate whether a limitation in maximal, uncoupled respiration due to loss of cytochrome *c* from the mitochondrial electron transport chain is an early functional biomarker of primed cells responding to ABT-737. Consistent with our predictions, cell-based respiration assays rapidly identified a primed for death state in BCL-2 overexpressing MCF10A cells by measuring a cyt *c*-reversible attenuation of maximal O_2_ consumption. Bioenergetics-based profiling assays were then successfully extended to cancer cells, identifying a primed for death state in two human B-cell lymphoma cell lines.

**Figure 3:**
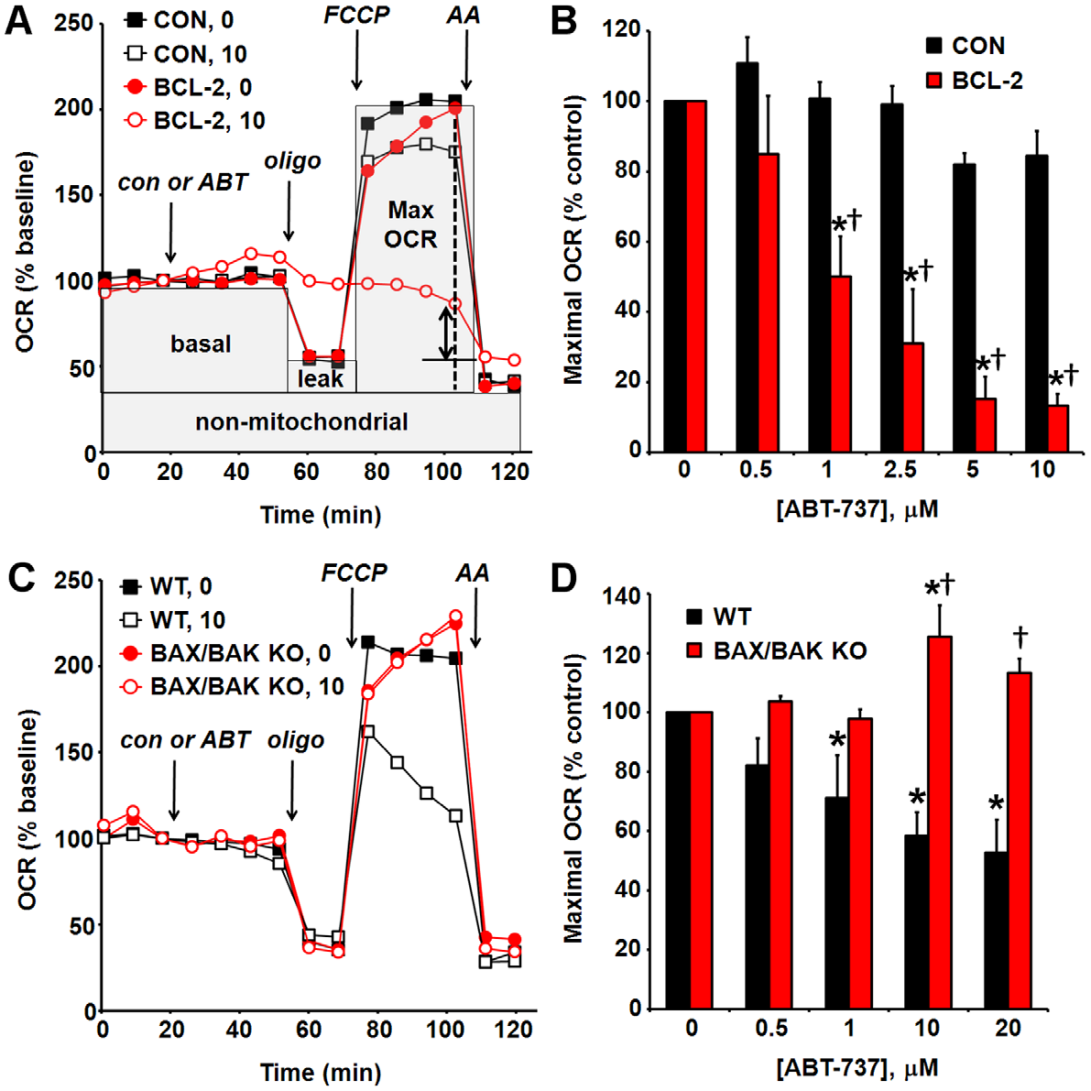
ABT-737 induces a BAX/BAK-dependent impairment of maximal O_2_ consumption rate in sensitive cells. (A) Representative bioenergetic profiles of MCF10A control-transfected (CON) and BCL-2 overexpressing (BCL-2) cells treated with vehicle (con) or ABT-737 (ABT, 10 µM), oligomycin (oligo, 0.5 µg/ml), FCCP (1 µM), and antimycin A (AA, 10 µM) as indicated. Pyruvate (10 mM) was added in combination with FCCP to ensure that substrate supply was not rate-limiting for maximal O_2_ consumption. The dotted line indicates the time point used to calculate maximal OCR. The solid line with arrows illustrates the reduction in MCF10A BCL-2 maximal OCR with ABT-737. Representative traces are means from one experiment performed in triplicate. OCR is baseline-normalized to the point prior to vehicle or ABT-737 addition. (B) Maximal OCR following addition of ABT-737 as % vehicle control (see A). Results are mean ± SE of 3–4 experiments with 2–5 replicates per experiment. *p<0.05 for ABT-737-treated relative to control-treated; †p<0.05 for MCF10A BCL-2 relative to control-transfected cells. (C) Representative bioenergetic profiles of immortalized wild type (WT) and BAX/BAK knockout (KO) mouse embryonic fibroblasts (MEF) treated with vehicle (con) or ABT-737 (ABT, 10 µM), oligo (0.2 µg/ml), FCCP (1 µM, WT, 2 µM KO), and AA (1 µM) as indicated. Traces are means from one experiment with 4–5 replicates. OCR is baseline-normalized as in A. (D) Maximal OCR following addition of ABT-737 as % vehicle control. Results are mean ± SE of 3 experiments with 2–5 replicates per experiment. Maximal OCR was determined as in A and B. Optimal oligomycin and FCCP concentrations were determined by titration for each cell type. *p<0.05 for ABT-737-treated relative to control-treated; †p<0.05 for BAX/BAK KO MEF relative to WT.

## Results

### Stable BCL-2 Overexpression in MCF10A Cells Induces an ABT-737-sensitive Primed for Death State

The ability of ABT-737 to induce apoptosis was measured to investigate whether stable BCL-2 overexpression in non-tumorigenic MCF10A human mammary epithelial cells [30] leads to a BCL-2 dependent primed for death state. BCL-2 overexpression sensitized MCF10A cells to induction of cell death by the BCL-2 inhibitor ABT-737 (Fig. 1A–C) although it conferred resistance to caspase-dependent apoptosis induced by other stimuli [30]. Cell death induced by ABT-737 in MCF10A BCL-2 overexpressing cells (MCF10A BCL-2) was characterized by early phosphotidylserine exposure, a hallmark of apoptosis detected by combined Annexin V and propidium iodide (PI) staining (Fig. 1A, B). Significant Annexin V staining occurred in cells still negative for PI, a nuclear stain that can only enter cells once the plasma membrane has been compromised (Fig. 1A, bottom right quadrant, and Fig. 1B, expressed as a percentage of PI negative cells). ABT-737 (0.5–10 µM) also increased PI staining, suggesting that apoptosis had already progressed to completion by 4 h in a large number of cells (Fig. 1C). This rapid cell death is consistent with a primed for death state [14]. Importantly, both Annexin V staining and PI staining were completely inhibited by the caspase inhibitor Q-VD, confirming an apoptotic mechanism (Fig. 1B, C). Overexpression of BCL-2 in MCF10A cells did not cause a reduction in MCL-1 (Fig. 1D), excluding the possibility that a decrease in this ABT-737-resistant anti-apoptotic BCL-2 family protein was responsible for the increased sensitivity of MCF10A BCL-2 cells to ABT-737.

**Figure 4:**
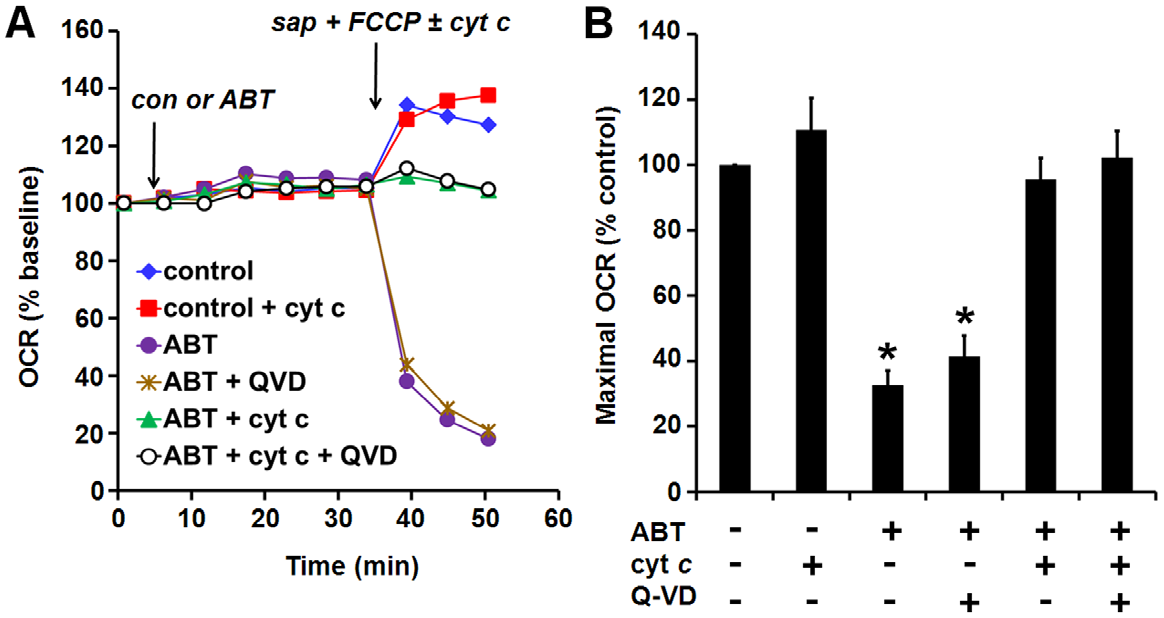
ABT-737-impaired maximal O_2_ consumption is rescued by exogenous cytochrome *c*. MCF10A BCL-2 cells were exposed to ABT-737 (10 µM) or vehicle (con) for 30 min, followed by acute plasma membrane permeabilization by saponin (sap, 10 µg/ml) in the presence of the complex II substrate succinate (5 mM), the complex I inhibitor rotenone (0.5 µM), the uncoupler FCCP (1 µM), and the presence or absence of cytochrome *c* (cyt *c*, 100 µM). The caspase inhibitor Q-VD (20 µM), when present, was added 30 min prior to ABT-737. (A) A representative experiment with treatments performed in triplicate. OCR is baseline-normalized to the point prior to vehicle or ABT-737 addition. (B) Quantification of the maximal OCR at the initial measurement point after permeabilization as a percentage of control (no ABT-737, Q-VD, or cyt *c*). Mean ± SE of 3–4 experiments with 2–3 replicates per experiment. *p<0.05 relative to the control treatment.

To test whether MCF10A BCL-2 cells were primed for cell death at the level of the mitochondrion, MCF10A BCL-2 cells and their control-transfected counterparts (MCF10A CON) were permeabilized by saponin and exposed to ABT-737. Selective plasma membrane permeabilization by saponin removes soluble cytoplasmic proteins and metabolites while preserving mitochondrial structure and function [31], [32]. Mitochondria within both cells lines released cyt *c* in response to the pore-forming peptide alamethicin (Fig. 2). However, mitochondria within BCL-2 overexpressing MCF10A cells were selectively vulnerable to ABT-737-induced cyt *c* release compared to MCF10A CON cells (Fig. 2). Similar results were obtained by exposing isolated mitochondria from MCF10A CON or MCF10A BCL-2 cells to ABT-737 (data not shown). The absolute dependence of BCL-2 overexpressing but not control cells on BCL-2 for mitochondrial cyt *c* retention and survival, as revealed by sensitivity to the BCL-2 antagonist ABT-737, indicates that these cells can be used to model the primed for death state that is a frequent characteristic of cancer cells.

**Figure 5:**
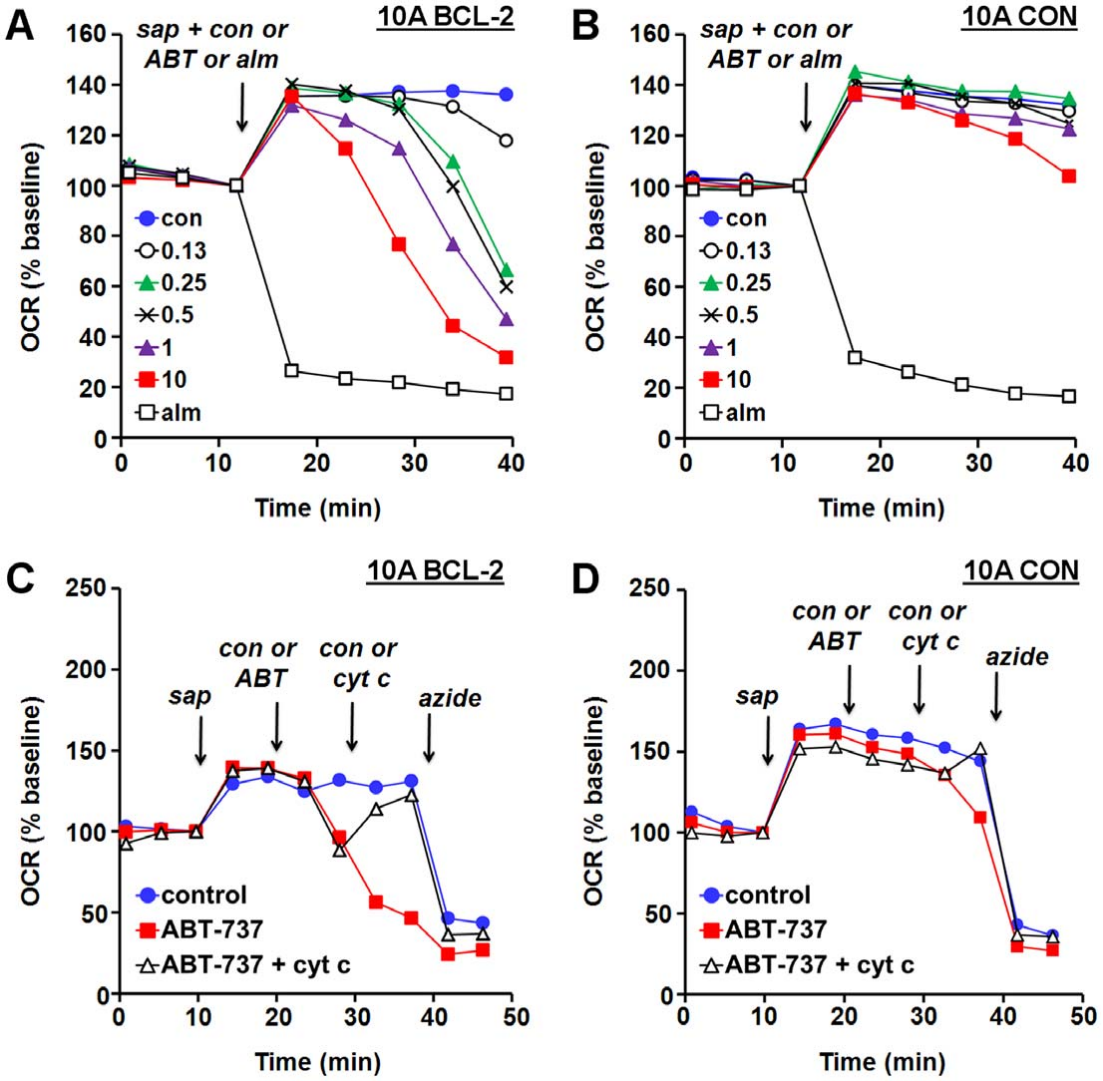
A respiration-based microplate assay detects cell death priming at the level of the mitochondrion. (A) MCF10A BCL-2 cells were exposed at the arrow to saponin (sap, 10 µg/ml) plus succinate (5 mM), rotenone (0.5 mM), ADP (1 mM), K_2_HPO_4_ (3.6 mM), and ABT-737 (ABT), vehicle (con), or alamethicin (alm, 80 µg/ml). Numbers in legend correspond to ABT-737 concentration in µM. (B) MCF10A CON cells receiving the treatments described in A. (C) MCF10A BCL-2 cells were exposed to saponin (10 µg/ml) plus succinate (5 mM), rotenone (0.5 mM), ADP (1 mM), and K_2_HPO_4_ (3.6 mM), followed by ABT-737 (ABT, 10 µM) or vehicle (con), cyt *c* (100 µM) or con, and sodium azide (5 mM). (D) MCF10A CON cells receiving the treatments described in C. All results are means from one experiment in triplicate and are representative of at least three independent experiments. OCR is baseline-normalized to the point prior to saponin addition.

### ABT-737 Induces a BAX/BAK-dependent Impairment of Maximal O_2_ Consumption Rate in Sensitive Cells

We next tested whether cell-based respiration measurements could be used as a functional biomarker for primed cells that respond to ABT-737 by releasing cyt *c* from the mitochondrial electron transport chain. A bioenergetic profile is obtained by successive addition of the F_0_F_1_ ATP synthase inhibitor oligomycin, the uncoupler FCCP, and an electron transport chain inhibitor such as antimycin A (Fig. 3A) [33], [34]. Oligomycin is used to help identify uncoupling, an off-target activity of several of the early BCL-2 inhibitors identified [35]. With the F_o_F_1_ ATP synthase blocked by oligomycin, O_2_ consumption decreases and the residual O_2_ consumption rate (OCR) is largely dependent upon proton (H+) leak across the inner membrane (Fig. 3A) [34]. The greater the H+ leak, the higher the OCR in the presence of oligomycin. The maximal OCR, or “respiratory capacity,” is revealed by uncoupling mitochondria using FCCP (Fig. 3A). FCCP makes the inner membrane freely permeable to H+. When electron transport is not limited by H+ diffusion through the ATP synthase, OCR increases to the maximum extent supported by the electron transport chain and substrate supply [34]. The final addition of antimycin A to inhibit the electron transport chain enables subtraction of non-respiratory O_2_ consumption (Fig. 3A).

**Figure 6:**
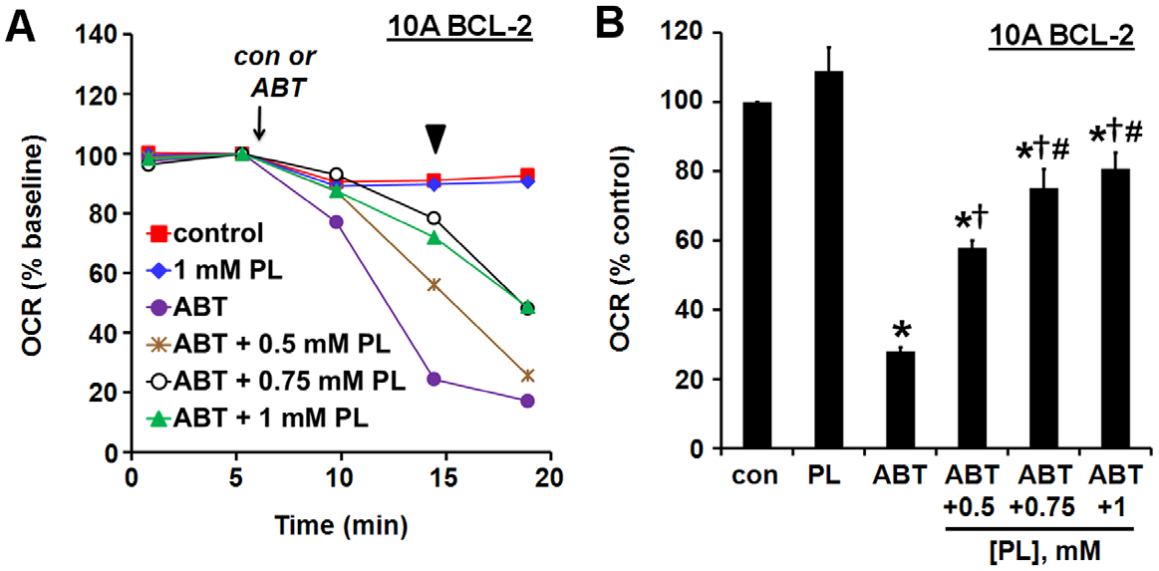
The BAX inhibitor propranolol delays the ABT-737-induced decline in respiration. (A) MCF10A BCL-2 cells were permeabilized as in Figure 5 in the presence or absence of propranolol (PL), and then exposed to ABT-737 (10 µM) or vehicle (con). Results are normalized to OCR just prior to ABT-737 or vehicle addition and are means from one experiment in triplicate. (B) Quantification of the experiment depicted in A. OCR at the second point after ABT-737 or con addition (arrowhead in A) is expressed as a percentage of control (no ABT-737 or PL). Mean ± SE of 3–4 experiments with 2–3 replicates per experiment. *p<0.05 relative to the control treatment; †p<0.05 relative to treatment with ABT-737 alone; #p<0.05 for 0.75 or 1 mM PL+ ABT-737 relative to 0.5 mM PL+ ABT-737.

Because cells have excess respiratory capacity, mitochondrial cyt *c* release is predicted to be rate-limiting for maximal OCR before basal OCR becomes inhibited [36]. We observed a dramatic dose-dependent impairment of maximal OCR in MCF10A BCL-2 overexpressing but not control-transfected cells treated with ABT-737 (Fig. 3A, B), correlating with the selective induction of cyt *c* release from mitochondria within MCF10A BCL-2 cells (Fig. 2). In addition, 10 µM ABT-737 significantly increased oligomycin-insensitive OCR in MCF10A BCL-2 cells (139±14%, n = 4 compared to vehicle treatment) but not in MCF10A CON cells (109±10%, n = 4 compared to vehicle treatment, Fig. 3A). This finding suggests that ABT-737 also has the ability to uncouple mitochondria and/or increase O_2_ consumption at sites other than cytochrome *c* oxidase (e.g. due to reactive oxygen species production) when BCL-2 is overexpressed. In the absence of ABT-737, the bioenergetic profile of MCF10A CON and MCF10A BCL-2 cells was similar (Fig. 3A). Baseline-normalized rather than absolute OCR values are presented to reduce variability due to well-to-well differences in plating density. However, determinations of maximal OCR and ABT-737-induced impairments were similar when expressing results in absolute OCR (Fig. S1).

**Figure 7:**
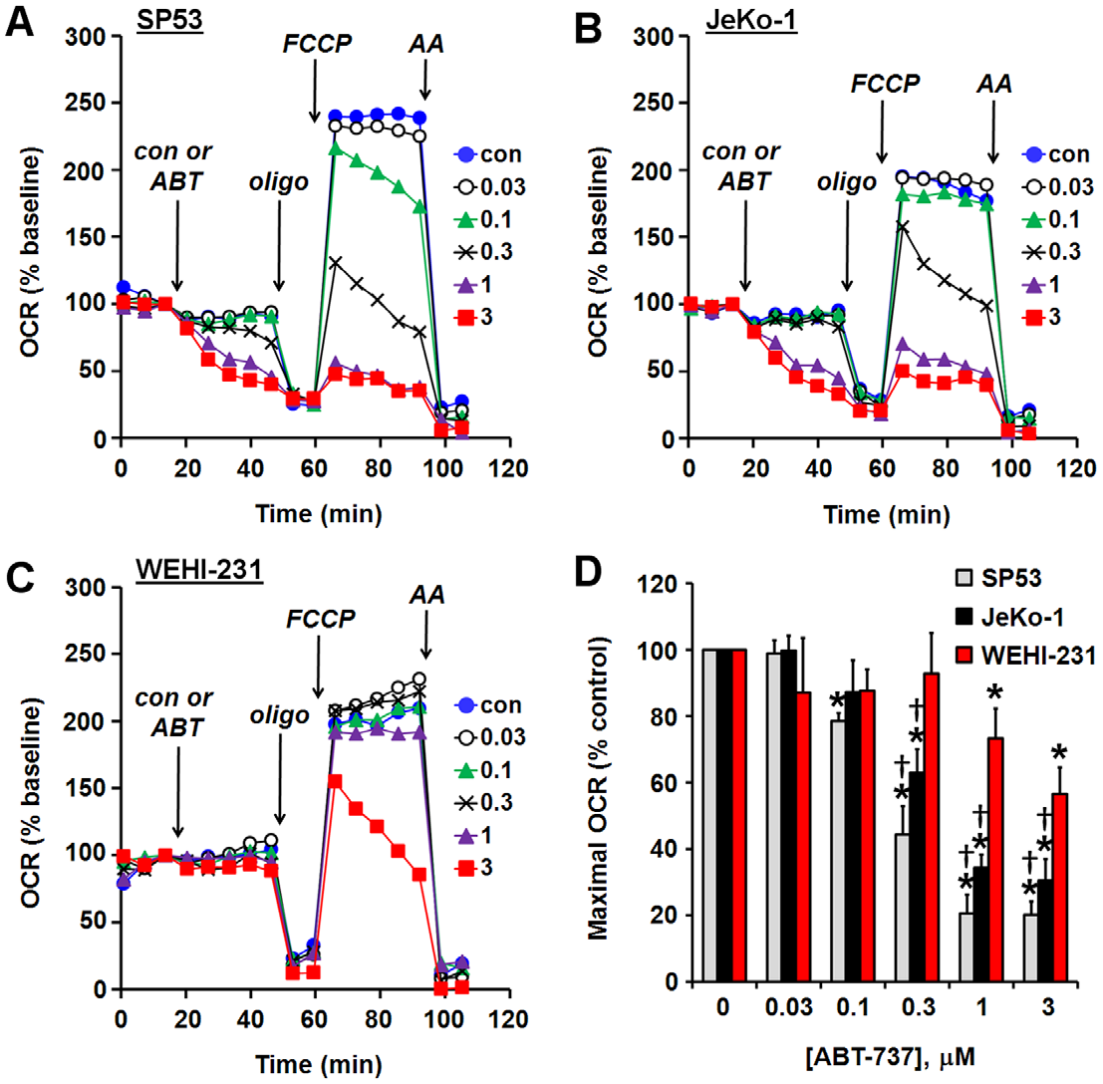
ABT-737 induces dose-dependent impairment of maximal O_2_ consumption rate in B-cell lymphoma cells. (A–C) Representative bioenergetic profiles of SP53 (A), JeKo-1 (B), and WEHI-231 (C) cells treated with vehicle (con) or ABT-737 (ABT), oligomycin (oligo, 0.3 µg/ml), FCCP (1 µM for SP53 cells, 3 µM for JeKo-1 and WEHI-231 cells), and antimycin A (AA, 1 µM) as indicated. Optimal oligomycin and FCCP concentrations were determined by titration for each cell type. Pyruvate (10 mM) was added in combination with FCCP. Numbers in legends correspond to ABT-737 concentration in µM. Representative traces are means from one experiment performed in triplicate. OCR is baseline-normalized to the point prior to vehicle or ABT-737 addition. (D) Maximal OCR following addition of ABT-737 as % vehicle control. Maximal OCR was determined as in Fig. 3. Results are mean ± SE of 3 experiments with 2–3 replicates per experiment. *p<0.05 for ABT-737-treated relative to control-treated; †p<0.05 relative to WEHI-231 cells treated with the same concentration of ABT-737.

It was previously shown that BAX or BAK is required for ABT-737-induced apoptosis by comparing BAX/BAK double knockout (KO) mouse embryonic fibroblasts (MEF) to wild type (WT) cells [37]. We measured the bioenergetic profile of both cell types to determine whether changes in respiration caused by ABT-737 are also BAX/BAK dependent. ABT-737 impaired the maximal OCR of WT MEF (Fig. 3C, D), although these cells were less sensitive to ABT-737 compared to MCF10A BCL-2 cells. In addition, uncoupled respiration in ABT-737-treated WT MEFs declined progressively over a 30 min period in contrast to control-treated cells (Fig. 3C). The progressive decline in uncoupled respiration and reduction of maximal OCR were prevented by BAX/BAK deficiency, consistent with the hypothesis that these bioenergetic signatures are due to BAX/BAK-dependent cyt *c* release. In contrast to MCF10A BCL-2 cells, a significant increase in oligomycin-insensitive OCR was not observed in WT or BAX/BAK KO MEF treated with 10 µM ABT-737 (108±7% (WT) and 99±4% (BAX/BAK KO) compared to vehicle treatment, respectively, n = 3, Fig. 3C), suggesting that this effect is specific to BCL-2 overexpression.

**Figure 8:**
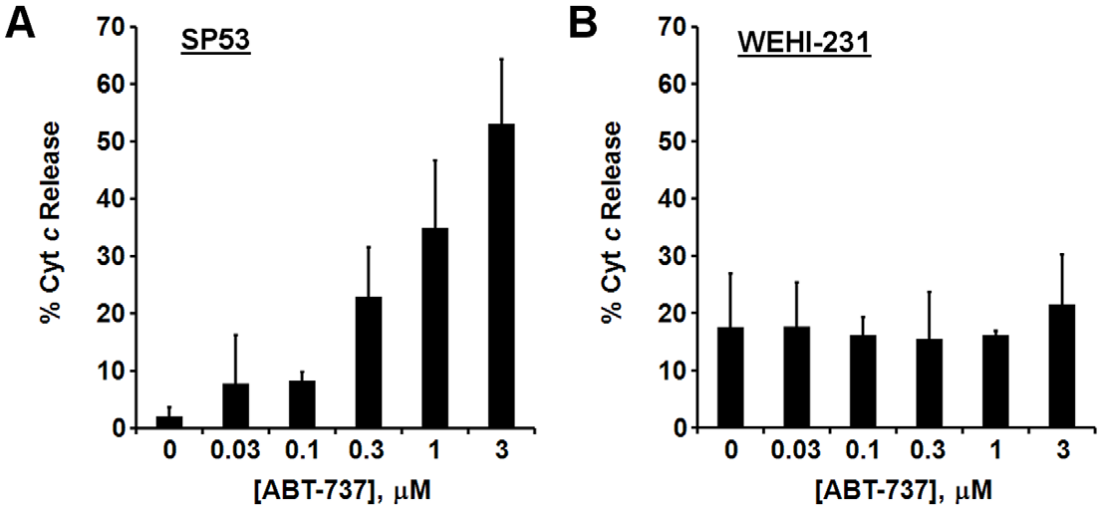
Dose-dependent cytochrome *c* redistribution in SP53 cells but not WEHI-231 cells treated with ABT-737. Saponin (10 µg/ml) was injected to liberate the contents of the cytoplasm into the assay medium after a 30 min incubation of intact cells with vehicle or ABT-737 (as indicated). Assays were conducted using the XF24 and the saponin injection corresponded to the time of oligomycin injection in Fig. 7. Cyt *c* was quantified by ELISA in the medium and cell lysate. Results are expressed as % cyt *c* released into the medium compared to the total cyt *c* that was quantified (mean ± SD, n = 3). (A) SP53 cells. (B) WEHI-231 cells.

### ABT-737-impaired Maximal O_2_ Consumption is Rescued by Exogenous Cytochrome *c*

To investigate whether maximal OCR impairments were due to loss of cyt *c* from the electron transport chain, we acutely permeabilized MCF10A BCL-2 overexpressing cells after ABT-737 treatment and tested whether addition of purified cyt *c* could restore maximal respiration supported by exogenous respiratory substrate (Fig. 4). The mitochondrial complex II substrate succinate was used for these experiments because evidence indicates that apoptotic caspase activity downstream of cyt *c* release can interfere with complex I activity [38]. Addition of saponin at a concentration that selectively permeabilizes the plasma membrane, in combination with FCCP and succinate to induce maximal OCR, led to respiratory stimulation in control-treated cells that was the same in the presence or absence of cyt *c* (Fig. 4A, B). The absence of a cyt *c* effect indicates that the mitochondrial outer membrane was intact in control-treated cells. In contrast, a rapid loss of O_2_ consumption was observed in ABT-737-treated cells. This loss was largely rescued by exogenous cyt *c* but not by the caspase inhibitor QV-D (Fig. 4A, B). The failure of Q-VD to rescue the respiratory deficit was not due to an off-target effect of Q-VD on respiration since Q-VD did not influence the ability of cyt *c* to rescue OCR in ABT-737-treated cells (Fig. 4A, B). These findings indicate that the large drop in OCR was due to loss of functional cyt *c* from the electron transport chain.

**Figure 9:**
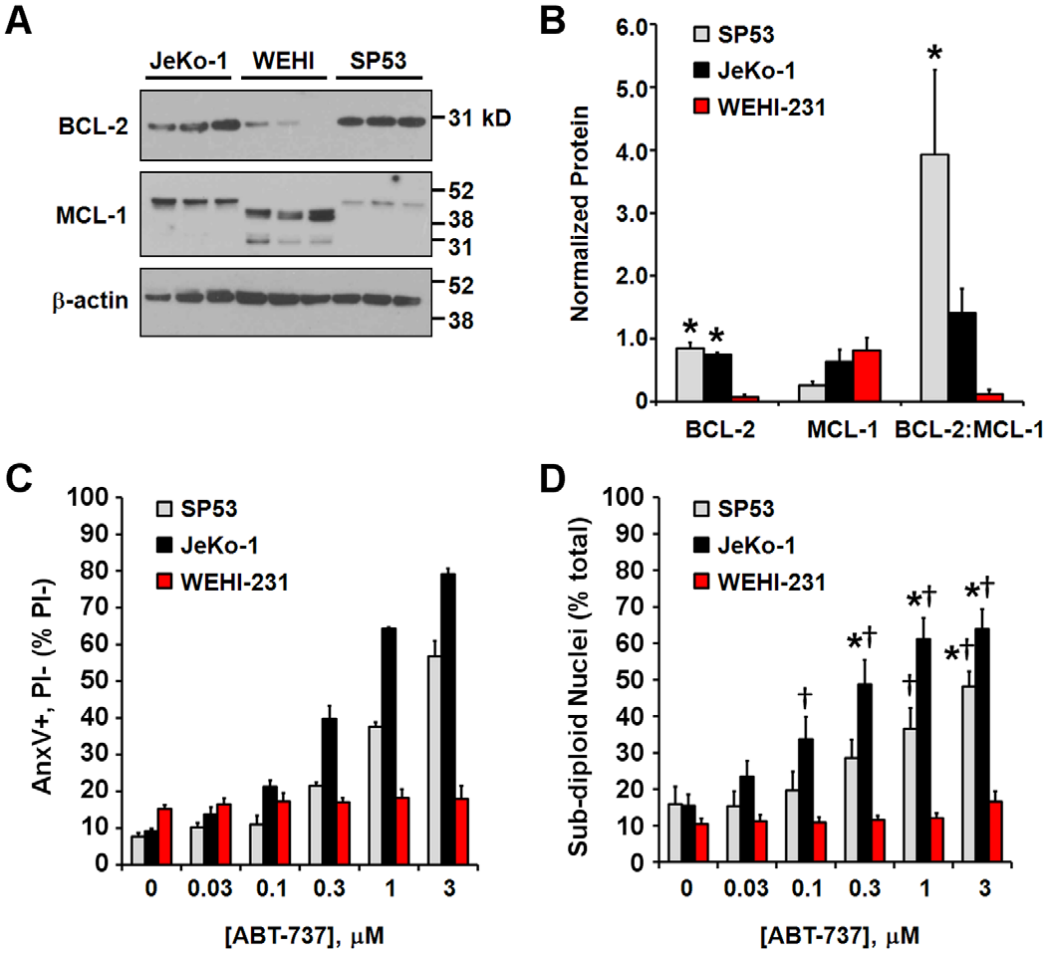
SP53 and JeKo-1 lymphoma cells exhibit a high BCL-2:MCL-1 ratio and a primed phenotype. (A) Expression levels of BCL-2 and MCL-1 relative to β-actin loading control in B-cell lymphoma cell lines. Mouse MCL-1 protein (WEHI-231) is known to migrate faster than the human form of the protein (SP53 and JeKo-1). (B) BCL-2 or MCL-1 protein levels normalized to β-actin and BCL-2:MCL-1 ratios following densitometric analysis of the immunoblots in A. Only the upper MCL-1 band corresponding to the full length protein was used for quantification. Results are mean ± SD, n = 3. *p<0.05 relative to WEHI-231 cells. (C) Early apoptosis, expressed as the percentage of PI negative cells that were AnxV-positive after 4 h of treatment with vehicle control or the indicated concentration of ABT-737. Results are mean ± SD of one experiment performed in triplicate. (D) Apoptosis, as determined by the percentage of sub-diploid nuclei. Results are mean ± SE of three (SP53) or five (JeKo-1 and WEHI-231) experiments performed in triplicate. *p<0.05 for ABT-737-treated relative to control-treated; †p<0.05 relative to WEHI-231 cells treated with the same concentration of ABT-737.

### A Respiration-based Microplate Assay Detects BAX/BAK-dependent Priming at the Level of the Mitochondrion

Next, we evaluated whether a respiration-based assay could also be used to detect cell death priming at the level of the mitochondrion. Addition of ABT-737 in combination with saponin and mitochondrial substrate resulted in a dose-dependent and progressive OCR reduction in MCF10A BCL-2 cells but not CON (Fig. 5A, B), likely reflecting the kinetics of cyt *c* release. Detection of released and retained cyt *c* by ELISA from the same cells used for XF24 assays confirmed that cyt *c* release was associated with changes in OCR (see Fig. 2).

**Figure 10:**
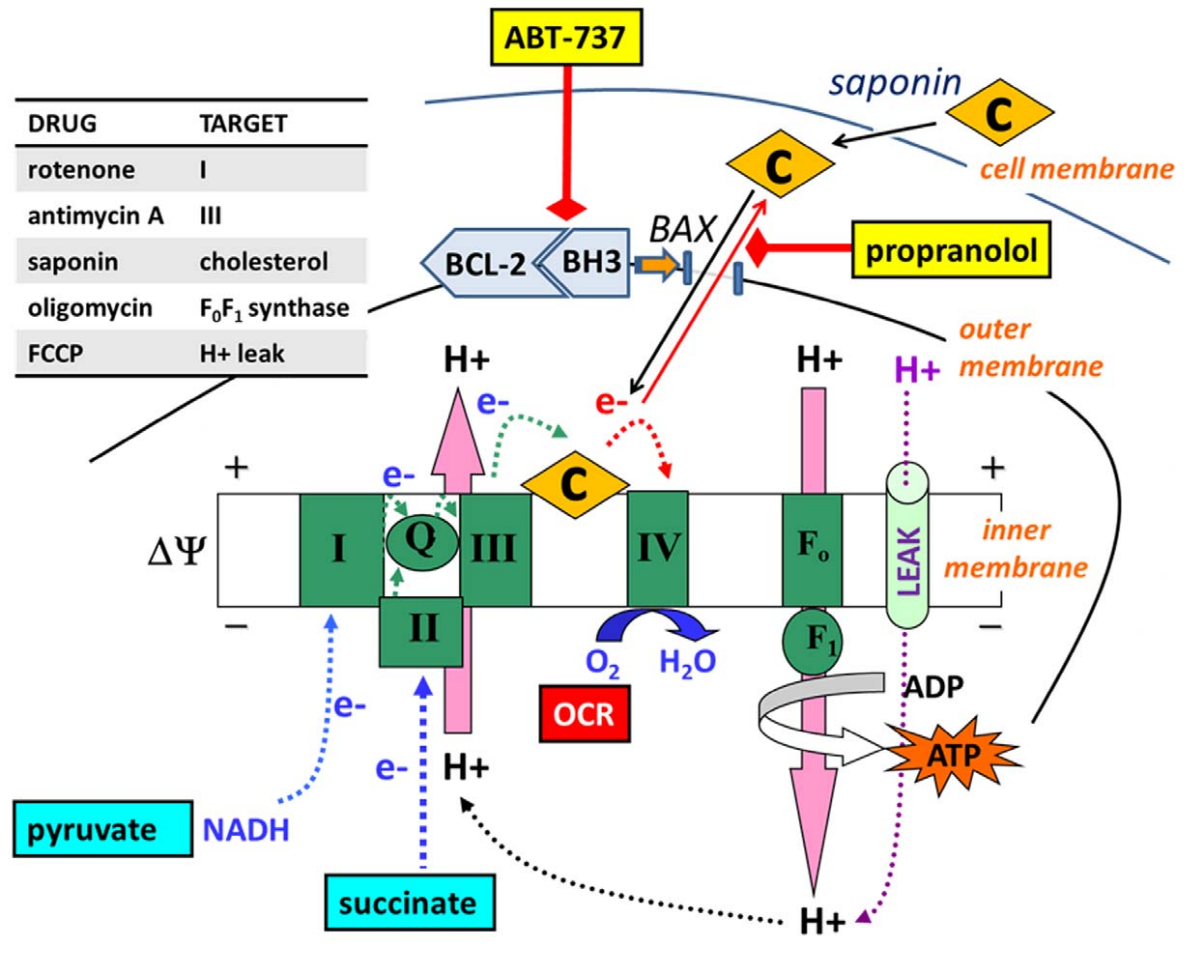
Bioenergetics-based profiling predicts BAX/BAK-dependent cytochrome *c* release. The schematic diagram and table (inset) outline drugs and drug targets used in the study. In bioenergetic-profiling assays, electrons are donated to the electron transport chain via complex I or complex II substrate (pyruvate or succinate, respectively). Cyt *c* (C), normally present in excess, is required to transfer electrons from complex III to complex IV. ABT-737 (or ABT-263) disrupts the binding of BCL-2 to BH3-only proteins (BH3) such as BIM on the mitochondrial outer membrane, enabling BAX/BAK activation, pore formation, and consequent cyt *c* release. The depletion of mitochondrial cyt *c* causes respiratory inhibition under conditions of high electron transport, such as in the presence of the uncoupler FCCP (Fig. 3–4,7) or a high ADP/ATP ratio (Fig. 5–6). Saponin permeabilizes the plasma membrane by extracting cholesterol, enabling the introduction of exogenous purified cyt *c*, which reverses respiratory inhibition due to cyt *c* release (Fig. 5). Propranolol, an inhibitor of BAX-induced cyt *c* release, delays the ABT-737-mediated loss of respiration (Fig. 6).

To demonstrate that respiration changes were indeed due to cyt *c* release, cells were permeabilized, and then exposed sequentially to ABT-737, cyt *c*, and sodium azide in the presence of succinate and ADP. ABT-737 caused a rapid loss of ADP-stimulated O_2_ consumption in permeabilized MCF10A BCL-2 cells that was acutely reversed by adding cyt *c* (Fig. 5C). Sodium azide inhibited cyt *c*-stimulated OCR, confirming that O_2_ consumption occurred at complex IV. In contrast to MCF10A BCL-2 cells, the OCRs of MCF10A CON cells were relatively stable following ABT-737 addition (Fig. 5D), as predicted by the resistance of MCF10A CON mitochondria to ABT-737-induced cyt *c* release (Fig. 2). However, the slight decline in OCR with 10 µM ABT-737 was also reversed by exogenous cyt *c*, consistent with ∼13% cyt *c* release (Fig. 2) at this high concentration of ABT-737. Cyt *c* co-treatment did not prevent respiratory impairment of intact cells exposed to ABT-737, indicating that a direct interaction of cyt *c* with ABT-737 was unlikely (data not shown).

We next took advantage of the fact that the amphiphilic membrane-active drug propranolol inhibits BAX-induced cyt *c* release from isolated mitochondria [39], [40] to test whether BAX/BAK dependence could also be established using microplate-based respirometry. Propranolol (PL, 0.5–1 mM) significantly delayed the ABT-737-induced decline in OCR (Fig. 6A, B), consistent with a BAX/BAK-dependent mechanism of ABT-737-induced mitochondrial outer membrane permeabilization. Collectively, the data in Fig. 5 and 6 show that it is feasible to detect drug-induced BAX/BAK-dependent cyt *c* release in primed cells by microplate-based respirometry. In addition, they demonstrate that ABT-737-mediated respiratory inhibition is not dependent on the presence of either FCCP or oligomycin since neither drug was added in these experiments.

### ABT-737 and ABT-263 Cause Impaired Maximal O_2_ Consumption and Apoptosis in Primed B-cell Lymphoma Cells

Encouraged by the ability of bioenergetics-based profiling to predict ABT-737 sensitivity in cells artificially primed by BCL-2 overexpression, we tested whether ABT-737 induces respiratory impairment in B-cell lymphoma cancer cells. The human mantle cell lymphoma cell lines SP53 (Fig. 7A, D) and JeKo-1 (Fig. 7B, D) displayed dose-dependent inhibition of maximal OCR starting at the relatively modest concentrations of 0.1 µM and 0.3 µM ABT-737, respectively. At higher ABT-737 concentrations (0.3, 1, and 3 µM in SP53 and 1 and 3 µM in JeKo-1), respiratory inhibition was evident even prior to the addition of FCCP. Respiratory inhibition was also apparent when examining absolute OCR; however as with other cell types, normalization of OCR reduced variability (Fig. S2). Quantification of cyt *c* redistribution in SP53 cells by ELISA confirmed that this respiratory inhibition was associated with cyt *c* release (Fig. 8A). In contrast to SP53 and JeKo-1 cells, WEHI-231 mouse B-cell lymphoma cells showed no inhibition of basal respiration and did not display an appreciable reduction in maximal OCR until a dose of 3 µM ABT-737 was reached (Fig. 7C, D). In addition, no ABT-737-induced cyt *c* redistribution was detected in WEHI-231 cells (Fig. 8B) although SP53 cells exhibited dose-dependent cyt *c* release at the same time point (Fig. 8A). The pore-forming peptide alamethicin induced >88% cyt *c* release from mitochondria within both cell types, demonstrating that cyt *c* release in WEHI-231 cells can be detected when it occurs (data not shown). ABT-263, the oral form of ABT-737 that is in clinical trials, induced a dose-dependent drop in SP53 OCR (Fig. S3A) but not in WEHI-231 OCR (Fig. S3B), similar to ABT-737.

Based on our respirometry results, we predicted that SP53 and JeKo-1 cells would have a high ratio of BCL-2 to MCL-1 compared to WEHI-231 cells and exhibit cell death priming. Consistent with this prediction, SP53 cells had the highest ratio of BCL-2 to MCL-1, followed by JeKo-1, and finally WEHI-231, which expressed little BCL-2 (Fig. 9A, B). SP53 and JeKo-1 cells, but not WEHI-231 cells, displayed dose-dependent apoptosis in response to ABT-737, as measured by Annexin V positivity/PI exclusion (Fig. 9C) and an increase in sub-diploid nuclei (Fig. 9D). No increase in PI staining was evident at this time point and annexin V staining was completely inhibited by Q-VD (data not shown), indicating caspase-dependent apoptosis. The relative differences in sensitivity of SP53, JeKo-1, and WEHI-231 cells to the orally bioavailable ABT-263 (Fig. S3C) were similar to the differences in sensitivity to ABT-737. Overall, our studies demonstrate proof-of-principle that microplate-based respirometry can be used to predict cyt *c* release and ABT-737 or ABT-263 apoptosis sensitivity in multiple cell types.

## Discussion

The BCL-2 anti-apoptotic pathway promotes cancer cell survival and can render tumor cells refractive to traditional chemotherapy [3], [5]. Although high affinity BCL-2 antagonists such as ABT-737/ABT-263 hold considerable promise for cancer therapy, complex factors dictate whether an individual tumor is likely to respond to treatment, including the levels and bound state of multiple anti- and pro-apoptotic BCL-2 family proteins [21]. Consequently we developed robust mechanism-based functional assays to rapidly identify cells primed to respond to ABT-737 based on the hypothesis that cyt *c* redistribution in cells limits the maximal rate of O_2_ consumption (Fig. 10). Crucial to the success of this approach, ABT-737 did not display BAX/BAK-independent uncoupling or respiratory inhibition (Fig. 3C, D), allowing for the detection of apoptosis-specific bioenergetic effects in cells.

Human MCF10A mammary epithelial cells are non-tumorigenic. Although immortalized, they are frequently used as a model for normal mammary epithelial cells [30], [41]. MCF10A cells were resistant to ABT-737 (Fig. 1), consistent with the low toxicity of ABT-737/ABT-263 to normal tissues [13], [16]. Stable BCL-2 overexpression primed MCF10A mammary epithelial cells for death (Fig. 1). We showed that the increased sensitivity was not due to a reduction in ABT-737-resistant MCL-1 (Fig. 1D), clearly demonstrating that the level of MCL-1 protein is an inadequate biomarker to predict ABT-737 sensitivity. Although elucidation of the priming mechanism was not an objective of this study, stable BCL-2 overexpression likely led to an accumulation of ABT-737-releasable BAX or BIM bound to BCL-2 at the mitochondria, consistent with other studies of primed cells [24], [42]–[44]. This may be in part due to inhibition of BIM degradation [24]. Importantly, an ABT-737-triggered impairment of maximal OCR was readily identified by microplate-based respirometry in primed MCF10A BCL-2 cells but not in unprimed control cells (Fig. 3, 4, 5), consistent with our hypothesis. Acute delivery of cyt *c* by selective plasma membrane permeabilization rescued respiration (Fig. 4), strongly suggesting that this impairment was due to mitochondrial cyt *c* release. However, the possibility that cyt *c* also has a direct action on ABT-737 or its target cannot be entirely excluded. The near complete loss of OCR following permeabilization of ABT-737-treated cells may have been due to dilution of the cytoplasm if cytoplasmic cyt *c* is able to maintain some degree of respiration following its release from mitochondria, as previously suggested [45]. Alternatively, saponin may have accelerated the process of BAX/BAK-mediated cyt *c* release in ABT-737-treated cells even though it did not affect the mitochondrial outer membrane permeability of control-treated cells [46].

Consistent with the sensitivity of BCL-2 overexpressing MCF10A cells to ABT-737, we found that B-cell lymphoma cells with relatively high BCL-2 levels (SP53 and JeKo-1 cells) were also ABT-737 sensitive (Fig. 9). SP53 cells were slightly more sensitive to ABT-737-induced respiratory inhibition compared to JeKo-1 cells (Fig. 7) although they were slightly less sensitive to apoptosis (Fig. 9). It is possible that differences in the apoptosis pathway downstream of cyt *c* release influenced the rate of cell death progression. In contrast to SP53 and JeKo-1 cells, WEHI-231 cells with minimal BCL-2 expression were resistant to ABT-737-induced apoptosis. Notably, JeKo-1 and WEHI-231 cells had similar levels of MCL-1 (Fig. 9A, B), further illustrating that MCL-1 level is not an accurate predictor of ABT-737 sensitivity. Although BCL-2:MCL-1 ratio was a reasonably good predictor of sensitivity in our limited pool of lymphoma cells, consistent with some studies [47], [48], microplate-based respirometry also predicted ABT-737 sensitivity, as well as sensitivity to the oral form ABT-263 (Navitoclax) that is the subject of multiple clinical trials. Large-scale comparisons of several methods will ultimately be necessary to determine whether our bioenergetics-based functional approach is a better prognostic indicator of ABT-737/ABT-263 sensitivity than established approaches.

In addition to reducing respiratory capacity, ABT-737 unexpectedly increased oligomycin-insensitive OCR in BCL-2 overexpressing cells but not in control-transfected cells, MEF cells, or B-cell lymphoma cell lines. This increase could be due to mitochondrial uncoupling or due in part to elevated reactive oxygen species production resulting from cyt *c* release [49], [50]. Because the increase in oligomycin-insensitive OCR was not observed in WT MEF cells or JeKo-1 or SP53 cells that still responded to ABT-737 with a significant reduction in maximal OCR, it is possible that this effect is specific to artificial BCL-2 overexpression. While investigation of the mechanism behind this increase was beyond the scope of the current study, this increase may serve as a secondary bioenergetic signature of primed cells if it occurs in some tumors that overexpress BCL-2.

ABT-737 synergizes with existing chemotherapeutics (e.g. taxanes) in many cases where it is ineffective as a single agent [19], [20]. The ability of cell-based respirometry to rapidly detect mitochondrial cyt *c* loss, an apoptotic checkpoint upon which drugs of diverse action converge, should facilitate the discovery of additional combination therapies that incorporate BCL-2 antagonists (e.g. by identifying drug combinations that prime cells to respond to ABT-737). The fact that this method uses intact cells is critical, since many drugs that synergize with ABT-737 do not act directly on mitochondria. In addition, by measuring oligomycin-insensitive OCR and by examining the cyt *c* reversibility of reductions in OCR, the method can rapidly eliminate novel drug candidates that display non-specific mitochondrial toxicity, a problem that plagued several of the early BCL-2 inhibitors identified [35], [37], [51], [52]. Because both BCL-2 overexpressing cells and BAX/BAK KO cells are highly resistant to apoptosis, it is important to note that sensitivity to ABT-737 does not result simply from apoptotic resistance, but depends on the specific genetic abnormality that caused the apoptotic resistance to develop. Since the specific underlying genetic mechanism will rarely be clearly defined in clinical samples, a primed for death bioenergetic signature could allow the susceptibility to ABT-737 to be predicted functionally without requiring the exact genetic abnormality to be known.

An additional microplate-based assay was developed in this study that uses permeabilized cells to detect cell death priming at the level of the mitochondrion. This method, which relies on a BAX-inhibitor suppressible, cyt *c*-reversible drop in OCR (Fig. 5, 6), may be useful for screening chemical libraries for drugs like ABT-737 that induce “mechanism-based” cyt *c* release without non-specific mitochondrial toxicity. The method can also be multiplexed to determination of cyt *c* release by ELISA (Fig. 2, 8) if confirmation of cyt *c* release is desired. However, this assay may prove less sensitive for the detection of priming compared to our intact cell method since cytoplasmic proteins that influence intrinsic apoptosis, e.g. BAX, will be reduced or lost following permeabilization. It is also possible that some cells are primed to respond to ABT-737, but not at the level of the mitochondrion (e.g. inhibition of non-mitochondrial protein-protein interactions in the intact cell may cause redistribution of pro-apoptotic BCL-2 family proteins to mitochondria). Such primed tumors, if they occur, would be correctly identified by our intact cell bioenergetic profiling method, but not by techniques that rely on isolated mitochondria or permeabilized cells.

A future goal is to adapt bioenergetics-based profiling for tumor biopsies. We have already successfully adapted microplate-based respirometry for brain tissue slices [33]. Mitochondrial function in tissue can be preserved for at least three hours *ex vivo* [33], providing a sufficient time window to assay for ABT-737/263 sensitivity. Direct tissue measurements would afford a significant advantage over cell death assays that require tumor dissociation and culture prior to treatment, which have the potential to change tumor cell properties. In addition, bioenergetics-based methods are far more rapid than assays based on cell death or protein quantification, with pronounced changes in SP53 and JeKo-1 cells seen in as little as 20 minutes of ABT-737 treatment (Fig. 7). The actual concentration of ABT-737/263 that elicits a robust bioenergetic response in either cell or tissue-based assays is not important as long as a predictive relationship between the response and the effect on tumor growth can be established. If successful, bioenergetics-based profiling of tumor biopsies could help advance ABT-263 or similar drugs through clinical trials by excluding patients unlikely to respond to treatment. The ultimate goal is to be able to use bioenergetic profiling to functionally identify the subset of patients with chemoresistant but primed tumors who can benefit from treatment that incorporates a BCL-2 antagonist.

## Materials and Methods

### Materials

ABT-737 was obtained from Abbott Laboratories (Abbott Park, IL) or purchased from ChemieTek (Indianapolis, IN). ABT-263 was purchased from ChemieTek. MCL-1 rabbit polyclonal antibody was purchased from Abcam (Cambridge, MA) or from Cell Signaling Technology, Inc. (Danvers, MA). BCL-2 mouse monoclonal antibody (clone 100) was from Millipore. β-actin mouse monoclonal antibody was obtained from Sigma-Aldrich (St. Louis, MO). Annexin V-FITC conjugate, human cytochrome *c* ELISA kit, and cell culture products were from Invitrogen (Carlsbad, CA). Rat/mouse cytochrome *c* ELISA kit was from R&D Systems (Minneapolis, MN). Other reagents were purchased from Sigma-Aldrich unless otherwise indicated.

### Cell Culture

Immortalized wild type (WT) and BAX/BAK deficient (BAX/BAK KO) mouse embryonic fibroblasts (MEF) were generously provided by Drs. Tullia Lindsten and Craig Thompson (University of Pennsylvania, Philadelphia, PA) [53]. MCF10A cells stably transfected with empty vector (pcDNA3) or vector overexpressing BCL-2 were previously described [30]. MEF cells were cultured in Dulbecco’s modified Eagle’s medium (DMEM) supplemented with 10% fetal bovine serum, L-glutamine (2 mM), penicillin (100 IU/ml), and streptomycin (100 µg/ml). MCF10A control-transfected and BCL-2 overexpressing cells were cultured in a 1∶1 mixture of DMEM and F12 medium (DMEM-F12) supplemented with 5% horse serum, hydrocortisone (0.5 µg/ml), insulin (10 µg/ml), epidermal growth factor (20 ng/ml), penicillin (100 IU/ml), and streptomycin (100 µg/ml). SP53, JeKo-1, and WEHI-231 B-cell lymphoma cell lines were cultured in RPMI 1640 medium supplemented with 5% fetal bovine serum, HEPES (10 mM), sodium pyruvate (1 mM), L-glutamine (2 mM), 2-mercaptoethanol (50 µM), penicillin (100 IU/ml), streptomycin (100 µg/ml), and 1X non-essential amino acids.

### Cell Death Measurements

Cells were treated with ABT-737, ABT-263, or vehicle (DMSO) for 4 h in XF24 assay medium (6×10^4^ MCF10A cells, see medium composition below) or RPMI 1640 medium (1×10^6^ B-cell lymphoma cells) and apoptosis was analyzed by Annexin-V-binding/PI exclusion or by sub-diploid nuclei determination as previously described [54]. FACS analysis was performed on Becton Dickinson FACScan or FACScalibur instruments (Becton Dickinson, San Jose, CA). Data analysis was performed with CellQuest software (Becton-Dickinson).

### XF24 Microplate-based Respirometry

O_2_ consumption measurements from intact and permeabilized cells were performed using an XF24 Extracellular Flux Analyzer (Seahorse Bioscience, Billerica, MA) as previously described [31], [55]. WT and BAX/BAK KO cells were plated at a density of 2×10^4^ to 4×10^4^ cells per well and MCF10A CON and BCL-2 cells were plated at a density of 4×10^4^ to 6×10^4^ cells per well to achieve ∼85% confluence at the time of assay (16–24 h after plating). All comparisons were made with cells at a similar density at the time of assay. One hundred percent confluence, which causes BAX upregulation in MCF10A cells [56], was avoided. Additionally, analysis was restricted to cells within a 15 passage window during which stable ABT-737-sensitivity was observed. XF24 assay medium for MCF10A cells and MEF cells consisted of 120 mM NaCl, 3.5 mM KCl, 1.3 mM CaCl_2_, 0.4 mM KH_2_PO_4_, 1 mM MgCl_2_, 5 mM HEPES, 15 mM glucose, and 4 mg/ml fatty acid free bovine serum albumin, pH 7.4. For experiments when cells were permeabilized, 1.3 mM CaCl_2_ was replaced by 1.86 mM CaCl_2_ plus 5 mM EGTA to yield a low Ca^2+^ assay medium that prevents mitochondrial Ca^2+^ overload. The free Ca^2+^ concentration of this assay medium is ∼100 nM which approximates [Ca^2+^] in the cytoplasm [57]. Results were similar when cells were assayed in normal assay medium and 5 mM EGTA, diluted from a pH-adjusted 500 mM stock, was included in the permeabilization solution (Fig. S4) [31]. Increased buffering capacity (20 mM HEPES) was used for low calcium experiments to help neutralize H+ released by the binding of Ca^2+^ with EGTA [58].

For the B-cell lymphoma cell lines SP53, JeKo-1, and WEHI-231 which grow in suspension, Cell-Tak (BD Biosciences) was used to attach 2×10^5^ cells per well to V7 microplates for respiration measurements. Plates were coated with Cell-Tak at 3.5 µg/cm^2^ of surface area, according to the recommendations of the manufacturer. The XF24 lymphoma assay medium for B-cell lymphoma cell lines consisted of bicarbonate-free DMEM (Seahorse Bioscience) supplemented with 11 mM glucose, 1 mM L-glutamine, 4 mg/ml fatty acid free bovine serum albumin, and 5 mM HEPES, pH 7.4. Saponin (10 µg/ml) was used to acutely permeabilize cells for measurement of cyt *c* redistribution. Succinate (5 mM), rotenone (0.5 µM), ADP (1 mM), K_2_HPO_4_ (3.6 mM) and EGTA (5 mM) were added to the permeabilization solution, which also contained 20 mM HEPES, to maintain mitochondrial respiratory function and prevent calcium-induced cyt *c* release.

For respirometry experiments, outliers which exhibited unstable OCR and/or a failure to respond to drug injections (>3 SD from the mean of other wells receiving the same treatment) were excluded from analysis. An example of an excluded outlier is shown in Fig. S5.

### Protein Detection by Immunoblot or ELISA

Cells were lysed in radioimmunoprecipitation assay (RIPA) buffer consisting of 150 mM NaCl, 50 mM Tris, 1 mM EDTA, 1 mM EGTA, 1% Triton X-100, 0.5% sodium deoxycholate, 0.1% sodium dodecyl sulfate (SDS), and Protease Inhibitor Cocktail Set III (EMD Biosciences), pH 7.4. Ten µg (MCF10A cells) or 70 µg (B-cell lymphoma cells) of protein was loaded on NuPAGE Novex 4–12% Bis-Tris gradient gels (Invitrogen). SDS-PAGE and immunodetection for BCL-2 (1∶5000), MCL-1 (1∶1000), and β-actin (1∶10,000) were performed as previously described [51]. Cyt *c* release was quantified using human (Invitrogen) or rat/mouse (R&D systems) commercial ELISA kits according to the instructions of the manufacturer. First, OCRs were measured using the XF24, cells were treated, and released cyt *c* was detected in XF24 assay medium following plasma membrane permeabilization by saponin. After the removal of XF24 assay medium, cells were lysed in 50 µl of RIPA buffer for the detection of retained cyt *c*.

### Statistics

One or two-way analysis of variance with repeated measures was employed to evaluate statistical significance, with p<0.05 considered significant. Tukey’s post-hoc analysis was used to compare individual groups. Statistical analyses were carried out using SAS version 9.2 statistics software (Cary, NC).

## Supporting Information

Figure S1 **Baseline normalization reduces the variability of O_2_ consumption rate measurements from MCF10A cells.** (A) and (B). MCF10A CON and MCF10A BCL-2 cells were exposed to two successive additions of FCCP (0.5 µM) followed by pyruvate (10 mM) and antimycin A (1 µM). Absolute (A) and baseline-normalized (B) OCR values are mean ± SD of one experiment performed in triplicate. Pyruvate increased uncoupled respiration and was therefore added in combination with FCCP in all subsequent experiments to ensure substrate supply was not rate-limiting for maximal O_2_ consumption. (C) and (D). MCF10A CON and MCF10A BCL-2 cells were exposed to successive additions of ABT-737 (10 µM), oligomycin (0.5 µg/ml), FCCP (1 µM) and antimycin A (10 µM). Absolute (C) and baseline-normalized (D) OCR values are mean ± SD of one experiment performed in triplicate. OCR in B and D is baseline-normalized to the third measurement point. Although the variability was higher for MCF10A BCL-2 cells compared to MCF10A CON in the individual experiments depicted here, this was unlikely to be related to BCL-2 overexpression since in other experiments the reverse was observed.
(TIF)

Figure S2 **Baseline normalization reduces the variability of O_2_ consumption rate measurements from B-cell lymphoma cells.** Absolute (A,C,E) and baseline-normalized (B,D,F) OCRs for the data in Fig. 7A–C, expressed as mean ± SD for experiments performed in triplicate. (A) and (B) SP53 cells. (C) and (D) JeKo-1 cells. (E) and (F) WEHI-231 cells.
(TIF)

Figure S3 **ABT-263 induces dose-dependent impairment of maximal O_2_ consumption rate in primed B-cell lymphoma cells.** (A–B) Representative bioenergetic profiles of SP53 (A) and WEHI-231 (B) cells treated with vehicle (con) or ABT-263, oligomycin (oligo, 0.3 µg/ml), FCCP (1 µM for SP53 cells, 3 µM for WEHI-231 cells), and antimycin A (AA, 1 µM), as indicated. Pyruvate (10 mM) was added in combination with FCCP. Numbers in legends correspond to ABT-263 concentration in µM. Representative traces are means from one experiment performed in triplicate and are representative of at least three independent experiments. OCR is baseline-normalized to the point prior to vehicle or ABT-263 addition. (C) Apoptosis, as determined by the percentage of sub-diploid nuclei. Results are mean ± SD from one experiment performed in triplicate and are representative of two (SP53), three (JeKo-1), or four (WEHI-231) independent experiments.
(TIF)

Figure S4 **Exogenous cytochrome** ***c*** **rescues ABT-737-impaired maximal respiration in MCF10A BCL-2 cells in normal assay medium.** MCF10A BCL-2 cells were exposed to ABT-737 (10 µM) or vehicle (con) for 30 min, followed by acute plasma membrane permeabilization by saponin (sap, 10 µg/ml) in the presence of the calcium chelator EGTA (5 mM), the complex II substrate succinate (5 mM), the complex I inhibitor rotenone (0.5 µM), the uncoupler FCCP (1 µM), and the presence or absence of cytochrome *c* (cyt *c*, 100 µM). The caspase inhibitor Q-VD (20 µM), when present, was added 30 min prior to ABT-737. Results are means from one experiment performed in triplicate. OCR is baseline-normalized to the point prior to vehicle or ABT-737 addition.
(TIF)

Figure S5 **Representative example of an excluded outlier.** Shown is the bioenergetic profile of immortalized BAX/BAK knockout (KO) mouse embryonic fibroblasts treated with vehicle (con) followed by oligomycin (oligo, 0.2 mg/ml), FCCP (2 µM), and antimycin A (AA, 1 µM). Pyruvate (10 mM) was added in combination with FCCP. Each trace represents data collected from an individual well of cells. Only three wells of a 24 well plate are shown for clarity. The outlier (filled squares) is denoted by an arrowhead. Outliers were infrequent and typically exhibited a steady decline in OCR from the first measurement, irrespective of drug additions, possibly due to cell damage during the washing step prior to the start of the assay.
(TIF)
