# Supplementary figures and images for: Rapid Detection of an ABT-737-Sensitive Primed for Death State in Cells Using Microplate-Based Respirometry
Source: PLoS One. 2012 Aug 3;7(8):e42487. doi: 10.1371/journal.pone.0042487 (PMC3411749; doi:10.1371/journal.pone.0042487)

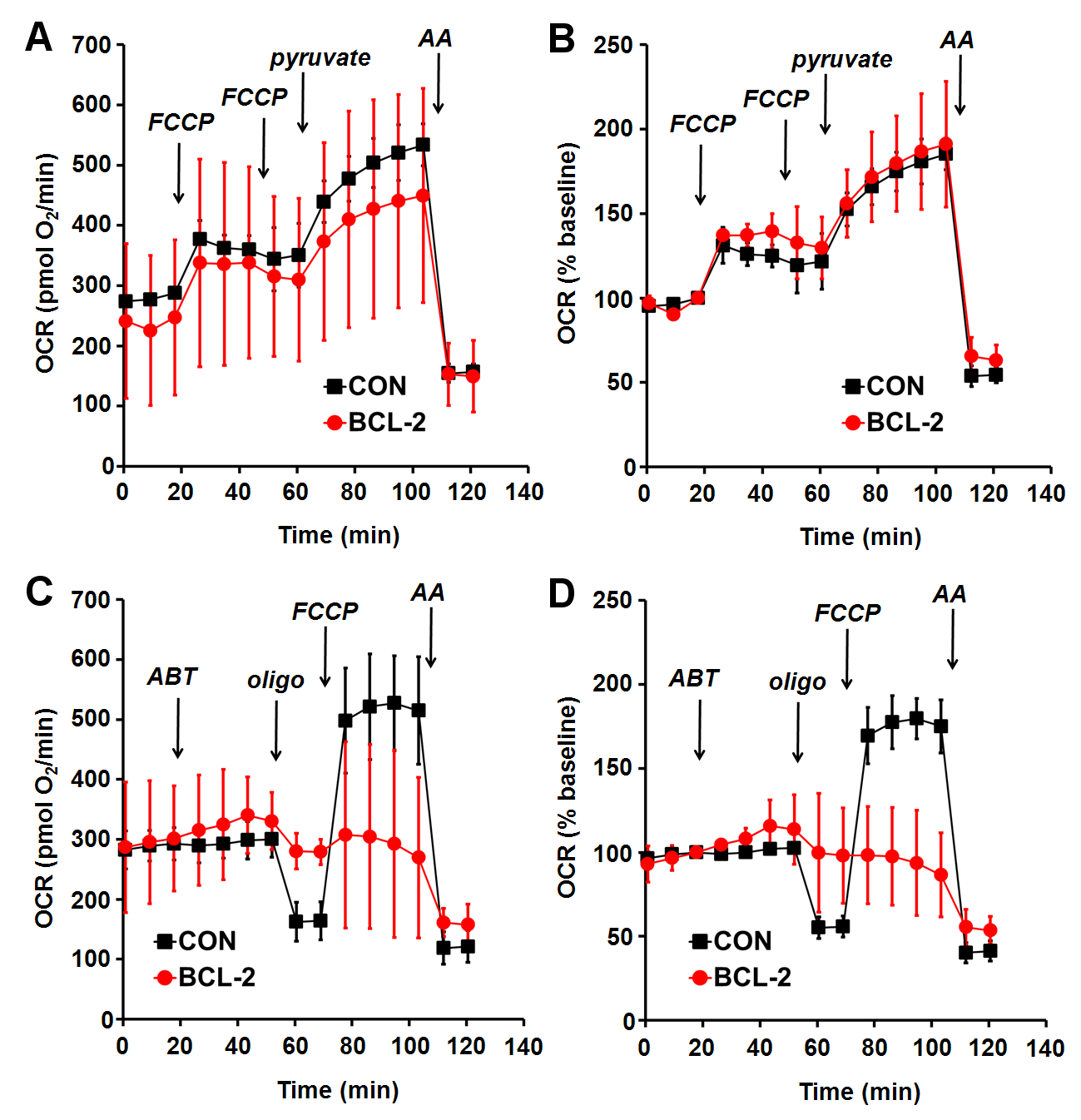

Supplement: Figure S1 — Baseline normalization reduces the variability of O2 consumption rate measurements from MCF10A cells. (A) and (B). MCF10A CON and MCF10A BCL-2 cells were exposed to two successive additions of FCCP (0.5 µM) followed by pyruvate (10 mM) and antimycin A (1 µM). Absolute (A) and baseline-normalized (B) OCR values are mean ± SD of one experiment performed in triplicate. Pyruvate increased uncoupled respiration and was therefore added in combination with FCCP in all subsequent experiments to ensure substrate supply was not rate-limiting for maximal O2 consumption. (C) and (D). MCF10A CON and MCF10A BCL-2 cells were exposed to successive additions of ABT-737 (10 µM), oligomycin (0.5 µg/ml), FCCP (1 µM) and antimycin A (10 µM). Absolute (C) and baseline-normalized (D) OCR values are mean ± SD of one experiment performed in triplicate. OCR in B and D is baseline-normalized to the third measurement point. Although the variability was higher for MCF10A BCL-2 cells compared to MCF10A CON in the individual experiments depicted here, this was unlikely to be related to BCL-2 overexpression since in other experiments the reverse was observed. (TIF) [file pone.0042487.s001.tif]

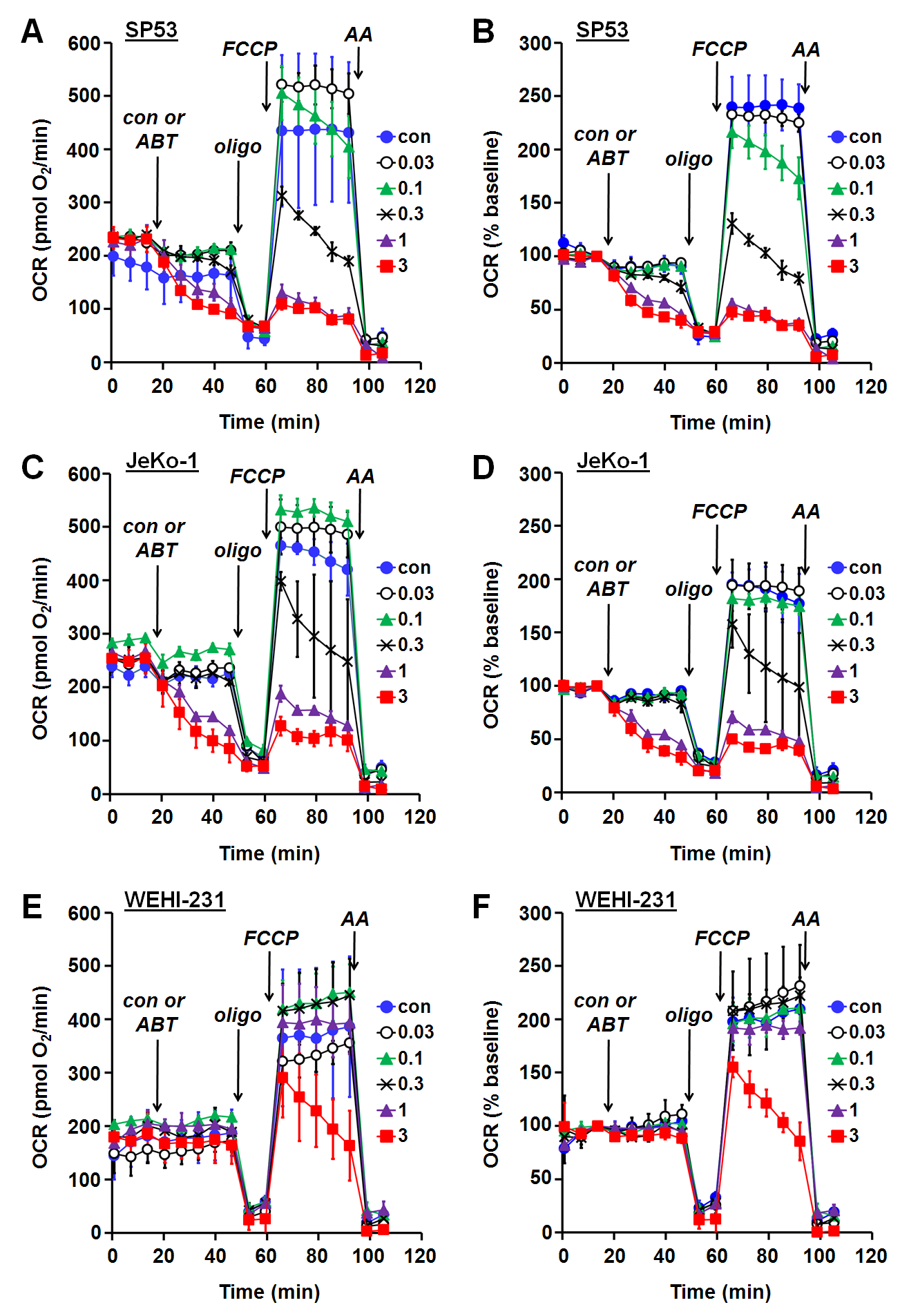

Supplement: Figure S2 — Baseline normalization reduces the variability of O2 consumption rate measurements from B-cell lymphoma cells. Absolute (A,C,E) and baseline-normalized (B,D,F) OCRs for the data in Fig. 7A–C, expressed as mean ± SD for experiments performed in triplicate. (A) and (B) SP53 cells. (C) and (D) JeKo-1 cells. (E) and (F) WEHI-231 cells. (TIF) [file pone.0042487.s002.tif]

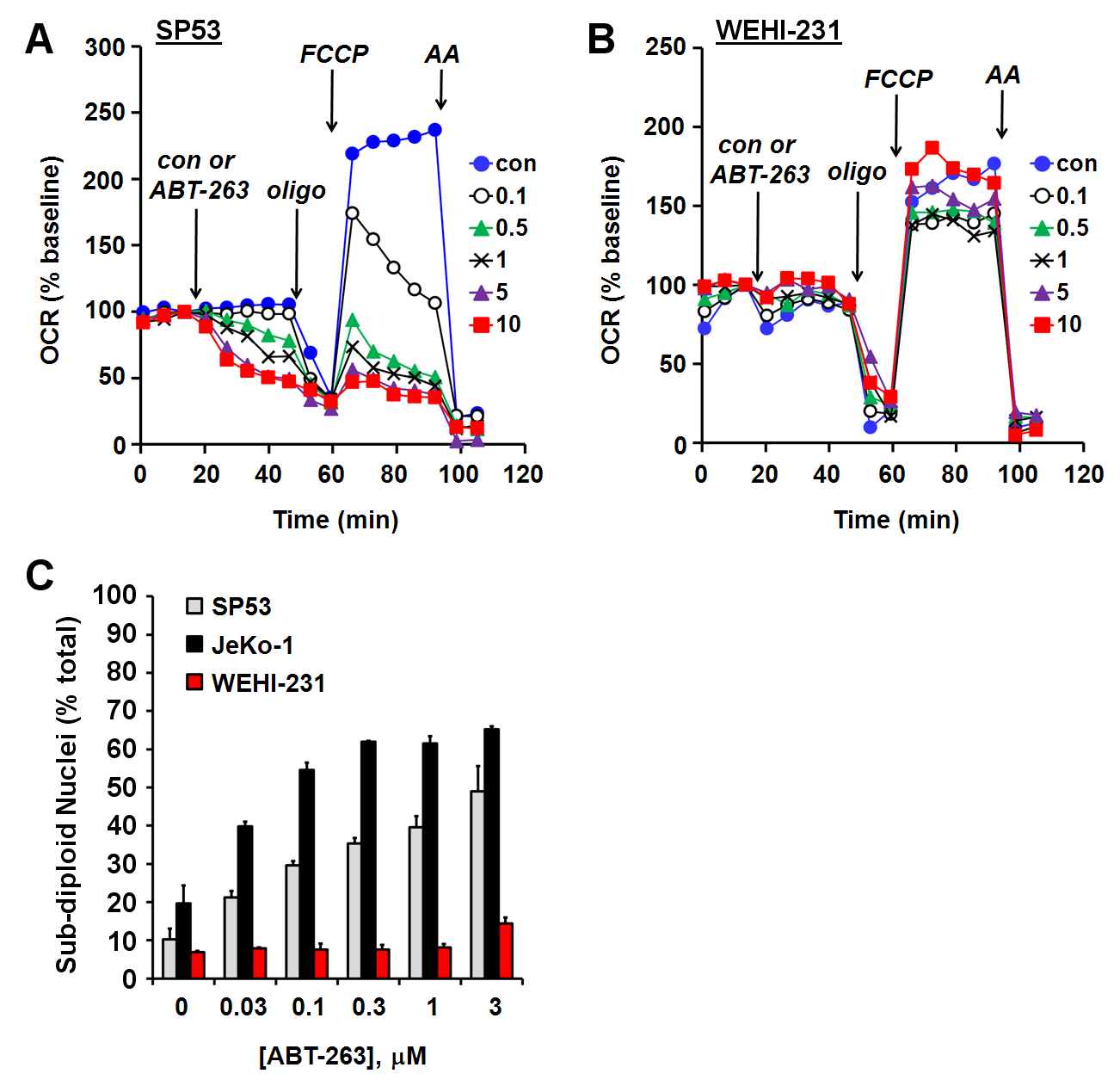

Supplement: Figure S3 — ABT-263 induces dose-dependent impairment of maximal O2 consumption rate in primed B-cell lymphoma cells. (A–B) Representative bioenergetic profiles of SP53 (A) and WEHI-231 (B) cells treated with vehicle (con) or ABT-263, oligomycin (oligo, 0.3 µg/ml), FCCP (1 µM for SP53 cells, 3 µM for WEHI-231 cells), and antimycin A (AA, 1 µM), as indicated. Pyruvate (10 mM) was added in combination with FCCP. Numbers in legends correspond to ABT-263 concentration in µM. Representative traces are means from one experiment performed in triplicate and are representative of at least three independent experiments. OCR is baseline-normalized to the point prior to vehicle or ABT-263 addition. (C) Apoptosis, as determined by the percentage of sub-diploid nuclei. Results are mean ± SD from one experiment performed in triplicate and are representative of two (SP53), three (JeKo-1), or four (WEHI-231) independent experiments. (TIF) [file pone.0042487.s003.tif]

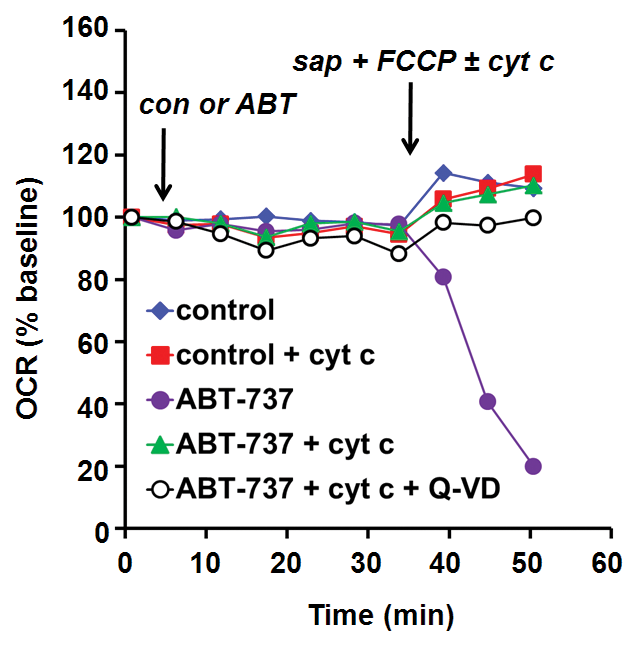

Supplement: Figure S4 — Exogenous cytochrome c rescues ABT-737-impaired maximal respiration in MCF10A BCL-2 cells in normal assay medium. MCF10A BCL-2 cells were exposed to ABT-737 (10 µM) or vehicle (con) for 30 min, followed by acute plasma membrane permeabilization by saponin (sap, 10 µg/ml) in the presence of the calcium chelator EGTA (5 mM), the complex II substrate succinate (5 mM), the complex I inhibitor rotenone (0.5 µM), the uncoupler FCCP (1 µM), and the presence or absence of cytochrome c (cyt c, 100 µM). The caspase inhibitor Q-VD (20 µM), when present, was added 30 min prior to ABT-737. Results are means from one experiment performed in triplicate. OCR is baseline-normalized to the point prior to vehicle or ABT-737 addition. (TIF) [file pone.0042487.s004.tif]

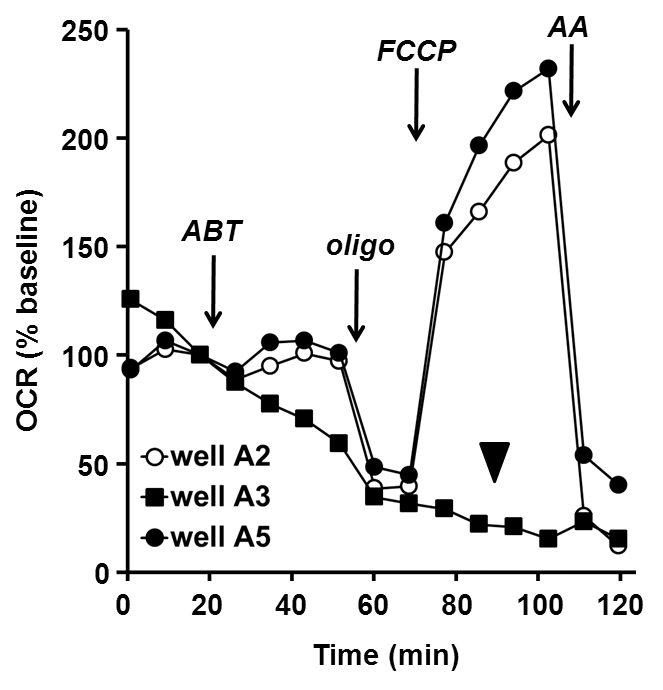

Supplement: Figure S5 — Representative example of an excluded outlier. Shown is the bioenergetic profile of immortalized BAX/BAK knockout (KO) mouse embryonic fibroblasts treated with vehicle (con) followed by oligomycin (oligo, 0.2 mg/ml), FCCP (2 µM), and antimycin A (AA, 1 µM). Pyruvate (10 mM) was added in combination with FCCP. Each trace represents data collected from an individual well of cells. Only three wells of a 24 well plate are shown for clarity. The outlier (filled squares) is denoted by an arrowhead. Outliers were infrequent and typically exhibited a steady decline in OCR from the first measurement, irrespective of drug additions, possibly due to cell damage during the washing step prior to the start of the assay. (TIF) [file pone.0042487.s005.tif]
